# Exposure to ambient air pollution and onset of Parkinson’s disease in a large cohort study

**DOI:** 10.1038/s41531-025-01156-z

**Published:** 2025-10-14

**Authors:** Babak Jahanshahi, Duncan McVicar, Neil Rowland

**Affiliations:** Queen’s Business School, Queen’s University Belfast, Belfast, UK

**Keywords:** Neurological disorders, Risk factors, Epidemiology

## Abstract

This population-based longitudinal cohort study examines the association between ambient air pollution (PM_2.5_ and NO_2_) and Parkinson’s disease (PD) using a 28% representative sample of Northern Ireland’s population (2009–2016). We matched complete address records to annual average PM_2.5_ and NO_2_ concentrations at a 1 km grid level and tracked PD onset via first receipt of PD medication. After controlling for confounding factors at individual, household, and neighbourhood levels, we found no association between medium-term PM_2.5_ or NO_2_ exposure and PD onset in the overall cohort, over-50s, or sex-stratified samples. However, a positive association was observed between PM_2.5_ exposure and PD onset in those under 50 in 2011, with weaker evidence for NO_2_. We discuss potential etiological and non-etiological explanations for this age-related difference.

## Introduction

The Global Burden of Disease (GBD) study reported that 8.5 million people globally had Parkinson’s disease (PD) in 2019, with cases growing due to aging populations, showing a 145% increase between 1990 and 2016^1^. In the United Kingdom (UK), PD prevalence for people over 20 in 2018 was 145,519, with a lifetime diagnosis risk of 2.7%. Incidence is expected to rise above 21,000 annually by 2025, alongside an 18% rise in prevalence^2^. In the United States (US) incidence rates for individuals aged 65+ range from 108 to 212 per 100,000, and from 47 to 77 per 100,000 for those aged 45+^3^. Understanding modifiable determinants of PD could help to mitigate this growing problem.

The etiology of PD remains unclear, with interactions between environmental and genetic factors implicated. Some emerging evidence links ambient air pollution, including particulate matter with a diameter of 2.5 micrometres or less (PM_2.5_) and nitrogen dioxide (NO_2_) to PD^4^. Ambient air pollution may contribute directly through neuro-inflammation and oxidative stress^5^ or indirectly through cardiovascular health^6–10^ or cerebrovascular disease^11^. Other risk factors include dairy products, pesticides, high body mass index, diabetes, cancer, and brain injury^12^.

The detrimental impact of air pollution on health in general has garnered significant attention in recent years^13,14^. Studies specifically on ambient air pollution and PD vary, examining pollutants such as PM_2.5_ and PM_10_^4,13,15–31^, as well as SO_2_, NO_2_, NOx, CO and Ozone^4,19,21,32–34^ and airborne metals such as lead, copper and manganese^17,35,36^.

Results are mixed, as evidenced by recent meta-analysis^20,29,37^, with some papers showing strong statistical associations^4,23,32,35,36^, while others have found weak or no associations^16,19,21,31,34,38^. This mixed picture is also reflected in the few studies that have estimated multipollutant models^4,30,39^. Variation in study context, exposure durations, and outcome measures likely contributes to these discrepancies^27,29^. For example, studies have restricted estimations to older age groups^19,26^ or younger^4,16^. Exposure over the short-term (<8 days)^23^, medium-term (≈1 year)^40^, and long-term (>2 years)^22^ has been analysed. Outcomes include self-reported PD cases, sometimes with verification from neurologists^16,21,31,38^; PD drug prescriptions^19,33^; cases from hospital or administrative databases^4,21–24,26,30,32,37^; and in one recent case, PD-related mortality collected from mortality registries^30^. But research findings have been mixed even within similar study types and contexts. Supplementary Table 1 in the Supplemental Content provides further details.

In this paper, we studied this question using new data from a large and nationally representative cohort tracked over an extended period. Specifically, we tracked a 28% representative sample of the Northern Ireland population between 2009 and 2016, with complete address records matched to annual average data on PM_2.5_ and NO_2_ concentrations at the 1 km grid-square level, with PD onset proxied by receipt of the first prescription for PD-related medication. Because studies suggest sex differences in PD pathophysiology (e.g., the role of oestrogen)^41^ and etiological differences in early versus late PD onset^42^, we also analysed associations by age and sex^38,40^. The study makes several distinctive contributions to the literature: it is the first to examine this association within Northern Ireland, a comparatively low-pollution context; our models account for delays in PD diagnoses, typically thought to range between 11 and 13 months^43^, but often overlooked in the existing literature; and it is one of very few studies to use prescription-based outcome measures or include subsample analyses by age and sex in the air pollution and PD literature.

## Results

Our main analysis sample was composed of a total of 292,925 individuals, from which 3089 started receiving medication for PD during the analysis period for our preferred model, i.e., at some point between 2012 semester 1 and 2016 semester 2 inclusive, or until such time as they attrited from the sample through death or emigration.

There were clear differences in observed 2011 characteristics between those who did and did not subsequently receive PD medication (see Table 1). For example, compared to those not experiencing PD onset over this period, those experiencing PD onset were more likely to be older; female; born in Northern Ireland; without educational qualifications; to report poor general health in the 2011 Census; to be inactive or unemployed; to be divorced/separated/widowed or never married; to have no dependent children in the household; to have no cars in the household; and to live in more deprived neighbourhoods. In other words, there was a clear age/sex/disadvantage contrast between the two groups. When comparing exposure to pollutants over the analysis period, those experiencing PD onset were found to be exposed to broadly similar levels of PM_2.5_ and NO_2_ on average compared to those not experiencing PD onset, with only slightly higher (lower) percentages in the highest (lowest) quartiles of the relevant exposure distributions.

**Table 1:** Characteristics and exposures of the study population by PD onset

	Without PD onset	With PD onset	Main analysis sample	Full NILS sample aged 28+ in 2011 census
Number of individuals Individual Characteristics (%)	289,836	3089	292,925	303,467
Age in 2011				
28–30	6.3	1.6	6.3	6.3
31–35	10.3	3.8	10.2	10.1
36–40	11.1	6.1	11.1	11.9
41–45	11.9	7.2	11.8	11.6
46–50	11.8	8.3	11.8	11.5
51–55	10.5	8.8	10.5	10.3
56–60	8.9	9.1	8.9	8.7
61–65	8.5	12.3	8.5	8.4
66-70	7.1	12.5	7.1	7.1
71–75	5.4	12.2	5.4	5.5
76–80	4.0	10.7	4.1	4.3
80+	4.2	7.4	4.3	5.2
Sex				
Male	47.5	40.3	47.4	47.3
Female	52.5	59.7	52.6	52.7
Country of birth				
Northern Ireland	88.1	91.4	88.1	87.7
Rest of the UK	5.2	4.7	5.2	5.3
Republic of Ireland	2.7	2.7	2.7	2.8
Born Elsewhere	3.9	1.4	3.9	4.2
Educational attainment				
No educational qualifications	32.3	54.1	33.0	33.3
Below degree or equivalent	37.7	28.0	37.1	36.9
Degree, equivalent or above	30.0	17.9	29.8	29.8
General health in 2011				
Very good/Good/Fair	92.1	80.7	92.0	91.5
Very Bad/Bad	7.9	19.3	8.0	8.4
Long term AL illness in 2011				
No long-term AL illness	71.9	41.6	71.6	70.4
Has a long-term AL illness	28.1	58.4	28.4	29.6
Economic activity in 2011				
Employed/Self-Employed	57.0	28.1	56.7	55.6
Inactive/Unemployed	43.0	71.9	43.4	44.4
Religion				
Catholic	38.5	36.8	38.5	38.3
Protestant/Other	46.7	50.9	46.7	46.8
No religion or none stated	14.8	12.3	14.8	15.0
Marital status in 2011				
Never married	19.6	13.8	19.6	19.9
Married	60.7	57.6	60.6	59.7
Separated/Divorced/Widowed	19.5	28.6	19.6	20.2
2011 household characteristics (%)				
Dependent children in HH				
0	64.2	80.8	64.4	63.8
1	14.1	8.9	14.0	13.7
2	13.5	5.9	13.5	13.2
3+	8.2	4.4	8.2	8.0
# of Cars in HH				
0	15.1	22.6	15.2	15.3
1	37.9	44.9	38.0	37.6
2	34.1	23.2	34.1	33.3
3+	12.8	9.1	12.7	12.4
# of persons per room in HH				
Max 1 person	97.9	98.5	97.9	96.6
More than 1 person	2.1	1.5	2.1	2.1
Neighbourhood characteristics (%)				
SOA deprivation (MDM) 2011				
1 (Most Deprived)	9.3	11.4	9.3	9.4
2	9.6	10.8	9.6	9.6
3	10.1	11.9	10.1	10.6
4	10.4	11.9	10.4	10.8
5	10.5	11.1	10.6	11.2
6	10.5	9.0	10.4	10.8
7	10.3	9.2	10.3	10.5
8	10.0	9.8	10.0	9.9
9	9.9	8.1	9.8	9.2
10 (Least Deprived)	9.4	7.8	9.4	8.0
Pollution exposures (µg/m³)				
PM_2.5_ (Mean[SD])	7.5 [1.5]	7.6 [1.5]	7.5 [1.5]	
NO_2_ (Mean[SD])	9.0 [4.7]	9.1 [4.6]	9.0 [4.7]	
PM_2.5_ quartiles (%)				
<6.49	25.6	24.6	25.6	
6.49–7.55	22.9	21.5	22.3	
7.55–8.83	31.4	31.5	31.4	
>8.83	18.0	20.3	18.0	
NO_2_ quartiles (%)				
<5.24	24.5	23.4	24.5	
5.24–8.10	25.0	24.9	25.0	
8.10–11.92	24.8	25.2	24.8	
>11.92	23.6	24.4	23.6	

The main analysis sample consists of all NILS members present in the 2011 Census who were aged 28 years or older at the time, had full address records, and were not in receipt of PD medication prior to January 2012, and not living in a communal establishment. The last column reports statistics for the full NILS sample aged 28+, returned at the 2011 Census, but similarly excluding those receiving PD medication prior to January 2012. The table reports the percentage of each sample reporting each characteristic in 2011. All variables are from Census 2011 except pollution exposures which are averaged over the whole exposure period. Pollutant quartiles are constructed using their distributions over the whole exposure period. Statistics are unweighted. MDM refers to the 2010 Multiple Deprivation Measure linked to 2011 Census address.

Table 2 presents our preferred Cox Proportional Hazard (CPH) model estimates for the effects of medium-term exposure to PM_2.5_ (Panel A) and NO_2_ (Panel B) on the onset of PD for the whole sample with 6-month exposures lagged 1 year. Model 1 estimates are unadjusted with no conditioning on measured characteristics, while Model 2 estimates are adjusted for measured individual, household and neighbourhood characteristics as listed in Table 1. Full results for Model 2 are presented in Supplementary Table 2.

**Table 2:** Hazard ratios (95% CI) for the association between medium-term exposure to PM_2.5_ and NO_2_ and Parkinson’s disease onset, 1 year lag, overall sample

	Model 1 (Unadjusted)		Model 2 (Adjusted)	
	(a) Linear	(b) Quartiles	(a) Linear	(b) Quartiles
Panel A (PM_2.5_, μg/m^3^)				
Linear model	1.03** (1.01–1.06)		0.99 (0.96–1.02)	
Quartiles model				
1st (reference)				
2nd		1.04 (0.93–1.16)		1.01 (0.90–1.13)
3rd		1.04 (0.92–1.17)		0.98 (0.87–1.11)
4th		1.18* (1.03–1.34)		1.01 (0.88–1.16)
Observations	2,688,153	2,688,153	2,688,153	2,688,153
Covariates	No	No	Yes	Yes
Panel B (NO_2_, μg/m^3^)				
Linear model	1.00 (1.00–1.01)		0.99 (0.98–1.00)	
Quartiles model				
1st (reference)				
2nd		0.95 (0.85–1.06)		0.96 (0.86–1.07)
3rd		1.00 (0.89–1.12)		0.95 (0.84–1.07)
4th		1.07 (0.96–1.19)		0.94 (0.83–1.06)
Observations	2,688,153	2,688,153	2,688,153	2,688,153
Covariates	No	No	Yes	Yes

Each model is a Cox Proportional Hazard (CPH) model with standard error clustered by SOA. Each cell presents the estimated hazard ratio for a 1 μg/m^3^ increase in PM_2.5_ (Panel A) and NO_2_ (Panel B) along with the 95% confidence interval in parentheses. The table reports a medium-term exposure effect defined as exposure to pollution over the semester with 1 year lag. Covariates are at individual, household and neighbourhood levels as listed in Table 1.

*p < 0.05; **p < 0.01; ***p < 0.001.

Despite some evidence of an association between PD onset and lagged PM_2.5_ exposure in the unadjusted models (Table 2, Model 1, Panel A), there was no such evidence in the models adjusted for differences in measured individual, household and neighbourhood factors (Table 2, Model 2), nor for NO_2_ exposure in either unadjusted or adjusted models. In the linear adjusted model, the estimated hazard ratios were 0.99 for a 1 μg/m^3^ increase in PM_2.5_ and 0.99 for a 1 μg/m^3^ increase in NO_2_ (95% confidence intervals 0.96–1.02 for PM_2.5_ and 0.98–1.00 for NO_2_), or 0.99 (0.92–1.06) for PM_2.5_ and 0.94 (0.89–1.00) for NO_2_ per IQR. Estimated Model 2 hazard ratios for exposure quartiles were also everywhere close to 1 and statistically insignificant for both pollutants, with no suggestion of dose response.

### Subsample analysis by sex and age

Tables 3 and 4 present equivalent estimates for the study population split by sex (Table 3), and age (Table 4), respectively. In each case, we present estimates for the linear and categorical exposure versions of the models in a single column to save space.

**Table 3:** Hazard ratios (95% CI) for the association between medium-term exposure to PM_2.5_ and NO_2_ and Parkinson’s disease onset, 1 year lag, by sex

	Male		Female	
	Model 1	Model 2	Model 1	Model 2
Panel A (PM_2.5_, μg/m^3^)				
Linear model	1.01 (0.97–1.06)	0.99 (0.95–1.04)	1.05** (1.01–1.08)	0.99 (0.95–1.03)
Quartiles model				
1st (reference)				
2nd	1.16 (0.97–1.39)	1.16 (0.97–1.39)	0.95 (0.82–1.11)	0.91 (0.79–1.05)
3rd	1.16 (0.96–1.40)	1.14 (0.94–1.38)	0.95 (0.81–1.12)	0.88 (0.75–1.04)
4th	1.17 (0.95–1.44)	1.09 (0.88–1.36)	1.16 (0.98–1.37)	0.95 (0.80–1.13)
Observations	1,265,787	1,265,787	1,422,366	1,422,366
Covariates	No	Yes	No	Yes
Panel B (NO_2_, μg/m^3^)				
Linear model	1.00 (0.99–1.01)	0.99 (0.98–1.01)	1.01 (1.00–1.02)	0.99 (0.98–1.00)
Quartiles model				
1st (reference)				
2nd	0.96 (0.81–1.14)	0.98 (0.82–1.17)	0.94 (0.81–1.08)	0.93 (0.80–1.07)
3rd	0.97 (0.82–1.14)	0.94 (0.81–1.18)	1.01 (0.88–1.16)	0.95 (0.82–1.10)
4th	1.01 (0.86–1.19)	0.94 (0.78–1.13)	1.10 (0.95–1.26)	0.93 (0.80–1.10)
Observations	1,265,787	1,265,787	1,422,366	1,422,366
Covariates	No	Yes	No	Yes

Each model is a Cox Proportional Hazard (CPH) model with standard error clustered by SOA. Each cell presents the estimated hazard ratio for a 1 μg/m^3^ increase in PM_2.5_ (Panel A) and NO_2_ (Panel B) along with the 95% confidence interval in parentheses. The table reports a medium-term exposure effect defined as exposure to pollution over the semester with 1 year lag. Covariates are at individual, household and neighbourhood levels listed in Table 1.

*p < 0.05; **p < 0.01; ***p < 0.001.

**Table 4:** Hazard ratios (95% CI) for the association between medium-term exposure to PM_2.5_ and NO_2_ and Parkinson’s Disease onset, 1 year lag, by age

	Age in 2011 <50 years		Age in 2011 ≥50 years	
	Model 1	Model 2	Model 1	Model 2
Panel A (PM_2.5_, μg/m^3^)				
Linear model	1.12*** (1.07–1.17)	1.05* (1.00–1.11)	1.00 (0.97–1.03)	0.97 (0.94–1.01)
Quartiles model				
1st (reference)				
2nd	1.32* (1.06–1.64)	1.26** (1.01–1.56)	0.94 (0.82–1.07)	0.93 (0.82–1.06)
3rd	1.33* (1.05–1.68)	1.23 (0.97–1.55)	0.93 (0.81–1.07)	0.91 (0.79–1.05)
4th	1.65*** (1.30–2.08)	1.30* (1.01–1.67)	1.02 (0.87–1.18)	0.93 (0.79-1.09)
Observations	1,413,737	1,413,737	1,274,416	1,274,416
Covariates	No	Yes	No	Yes
Panel B (NO_2_, μg/m^3^)				
Linear model	1.02*** (1.01–1.04)	1.00 (0.99–1.02)	0.99 (0.99–1.01)	0.99* (0.98–1.00)
Quartiles model				
1st (reference)				
2nd	1.01 (0.82–1.24)	1.01 (0.81–1.24)	0.93 (0.82–1.06)	0.94 (0.82–1.06)
3rd	1.31** (1.08–1.60)	1.24* (1.00–1.54)	0.89 (0.78–1.01)	0.86* (0.75–0.98)
4th	1.41*** (1.15–1.72)	1.19 (0.94–1.51)	0.95 (0.83–1.07)	0.86* (0.75–1.00)
Observations	1,413,737	1,413,737	1,274,416	1,274,416
Covariates	No	Yes	No	Yes

Each model is a Cox Proportional Hazard (CPH) model with standard error clustered by SOA. Each cell presents the estimated hazard ratio for a 1 μg/m^3^ increase in PM_2.5_ (Panel A) and NO_2_ (Panel B) along with the 95% confidence interval in parentheses. The table reports a medium-term exposure effect defined as exposure to pollution over the semester with 1 year lag. Covariates are at individual, household and neighbourhood levels listed in Table 1.

*p < 0.05; **p < 0.01; ***p < 0.001.

In line with Table 2, Table 3 shows no statistically significant associations between PM_2.5_ or NO_2_ exposure and PD onset for either men or women once estimates were adjusted for measured confounders, despite a statistically significant association between PM_2.5_ exposure and PD onset for females (but not males) in unadjusted models. Table 4 presents similar evidence for those aged 50+ years in 2011, with no statistically significant associations between pollution exposure and PD onset either in the adjusted or unadjusted models, bar a statistically significant hazard ratio marginally below 1 in the adjusted continuous model for NO_2_ exposure—statistically significant at the 95% level but not the 99% level—which likely reflected Type 1 error or residual confounding rather than a protective effect. In contrast, positive associations between the estimated hazard rate for PD onset and exposure to PM_2.5_ remained statistically significant at either the 95% or 99% level for the younger age group (under 50 years in 2011), after adjusting for measured confounders, in both the linear and categorical exposure models. The estimated hazard ratios were 1.05 (1.01–1.11) per 1 μg/m^3^ increase (1.13 (1.01–1.27) per IQR) in the adjusted linear exposure model and 1.26 (1.01–1.56), 1.23 (0.97–1.55) and 1.30 (1.01–1.67) for quartiles 2, 3 and 4 respectively, in the adjusted model with categorical exposures. There was also tentative evidence of a positive association with NO_2_ exposure, with the estimated hazard ratio for 3rd quartile exposure statistically significant at the 95% level (hazard ratio 1.24, confidence interval 1.00–1.54).

### Sensitivity analysis

Our finding of no significant association between PM_2.5_ or NO_2_ exposure and PD onset in the overall sample, after adjusting for confounders, was robust to extensive sensitivity analysis. This included: dual-pollutant models (Supplementary Fig. 1); imputing partially missing exposure data using within-individual average exposure in adjacent periods (Supplementary Table 3); extending the at-risk period back to 2010 semester 2 (Supplementary Table 4); varying the exposure lag between 0 and 18 months with the first at-risk period fixed at 2012 semester 1 (Supplementary Tables 5–7); increasing the exposure lag to 18 and 24 months by varying the first at-risk period while keeping the first exposure period fixed at 2011 semester 1 (Supplementary Tables 8 and 9); replacing six-monthly exposures with 2-year moving averages (Supplementary Tables 10–12); adopting an alternative onset definition requiring prescriptions in at least two consecutive semesters (Supplementary Table 13); re-estimation as a stratified Cox model (by sex, age and education) to explore sensitivity to violations of the proportional hazards assumption suggested by Schoenfeld residual testing (Supplementary Table 14); and either dropping the covariates for general health and limiting long-term illness (Supplementary Table 15) or supplementing these with additional covariates for chronic illness as of 2011 semester 1 or prescription-based measures for diabetes and cardio-vascular disease as of 2011 semester 1 (Supplementary Table 16).

We also investigated age-related differences in associations by lag length, re-estimating age-specific models with no lag, a 6-month lag, and an 18-month lag in pollution exposure (Supplementary Tables 17–19). Even for the younger age group, most significant associations disappeared with shorter lags (Supplementary Tables 17 and 18), consistent with diagnosis delay per prior evidence^43^. Results were consistent with our primary model (Table 3) when extending the lag to 18 months (Supplementary Table 19), showing null associations for the older group and significant positive associations for younger individuals, with some evidence of dose-response for both pollutants. For older individuals, no positive association was found even with longer lags, suggesting age-related differences in delays in diagnosis or prescription do not explain the age-group contrast. In addition, robustness tests on age-group-specific models using 2-year moving averages with a 1-year lag (Supplementary Table 20) and extending the analysis window to earlier periods (Supplementary Table 21) yielded broadly consistent results. Estimated PM_2.5_ effects remained similar to those in Table 3, while NO_2_ effects were no longer statistically significant in these additional analyses. Finally, estimated pollution effects for the younger age group were no longer statistically significant at the 95% level when using the alternative two-consecutive-period-prescription onset measure, although key estimated HRs were similar in magnitude (e.g. an estimated hazard ratio of 1.06 (0.99–1.14) per 1 μg/m^3^ increase in PM_2.5_ (see Supplementary Table 22)).

## Discussion

Our analysis aligns with prior studies that suggest a positive unadjusted association between medium-term PM_2.5_ exposures and PD onset^23–25^. However, pre-onset differences in characteristics such as age, sex and social disadvantage necessitated statistical adjustment, after which no positive associations with exposure to PM_2.5_ (nor NO_2_) were found, consistent with around half of the existing studies listed in Supplementary Table 1^15,16,21,31^.

Comparing our own findings to those of other studies is complicated by variation in factors such as exposure levels and durations studied, the extent to which exposures are lagged, PD measures employed, population studied, and the extent of statistical adjustment for potentially confounding measurable factors. For example, our study took place in a relatively low pollution context compared to most existing studies reported in Supplementary Table 1. Exceptions to this include Salimi et al.^21^, which similarly found no statistically significant association between exposure to both NO_2_ and PM_2.5_ and self-reported PD in New South Wales, Australia; Rumrich et al.^15^ which similarly found no association between PM_2.5_ exposures and PD onsets in Finnish data; and Cole-Hunter et al.^30^ which found a positive and statistically significant association between PD mortality and PM_2.5_ exposure across six European countries, in contrast to our own finding. Note, however, that Salimi et al.^21^ and Rumrich et al.^15^ used diagnosis-based outcome measures (closer to our own prescriptions-based measure), whereas Cole-Hunter et al.^30^ used a measure of PD mortality.

Among the few studies in the overall pollution-PD literature that have used prescription-based outcome measures, conclusions appear similarly mixed. For example, Cerza et al.^19^ (who combine drug registry data with other outcome measures) found no statistically significant association between PD and PM_2.5_ exposure. In contrast, Lee at al.^33^ reported a statistically significant positive association, albeit for pollutants other than PM_2.5_ and NO_2_ (e.g., CO and NOx).

This study is one of few to have examined evidence for heterogeneity in the effect of exposure to pollution on PD onset. In line with our estimates for the overall cohort, we found no evidence of PM_2.5_ or NO_2_ effects on PD onset in subsamples by sex and for those aged 50+ years based on Census 2011. But we found some evidence suggesting a positive and statistically significant association between PM_2.5_ and PD onset among those aged under 50 years at that point, with a more tentative (and less robust) indication of an association with NO_2_ exposure. Again, existing evidence in these respects is mixed. By sex, Lee et al.^40^ report a significant association with PM_2.5_ exposure for males but not females, and Liu et al.^38^ report no significant association for either males or females (although they report a significant association with PM_10_ exposure for females but not males). These two studies took contrasting approaches in other respects, however, with the former paper using hospital admission records in cohort data and the latter using self-reported measures in case-control data, for example. Lee et al.^40^ also estimated associations by age group, reporting a significant association with PM_2.5_ exposure among study participants over-65 but not among those under-65. Though not directly comparable, the former finding appears at odds with our own finding of a significant association for under-50s but not for over-50s.

Our finding—unique in the literature to date—that exposure to pollution (particularly PM_2.5_ pollution) is associated, albeit tentatively, with the onset of PD among <50s but not with onset of PD among those aged 50+ years in the NILS-EPD cohort might reflect a genuine difference in the etiology of PD across age cohorts. There is some existing evidence for etiologic differences in early versus late onset of PD^42^. However, given that PD has several clinical subtypes, pathogenic genes and putative causative environmental agents^44^, reaching a fuller understanding of such differences remains a challenge for the wider literature.

There may also be one or more non-etiological explanations for this contrast. We conjecture here that one such potential explanation is differences in the delay between onset of symptoms and PD diagnosis and/or first prescription for PD medication, given existing evidence of higher diagnostic delays for PD at older ages. Such delays introduce uncertainty regarding the relevant lag structure for exposures within models of PD onset and, crucially, might also differentially attenuate estimated associations at a given lag length by age group. Most of the PD-pollution literature ignores this potential source of bias^4,16,19,21,23,38^, although it is acknowledged by some studies^25,26^. In the absence of data on the onset of symptoms, we have been unable to examine this issue explicitly here. Nevertheless, we have shown that our conclusions, and crucially the contrast between estimated pollution effects for under-50s and over-50s, are robust to extending the lag of exposures beyond 12 months, although less so to shortening the lag below 12 months. The suggestion is that diagnostic delays and/or gaps between diagnosis and first prescription are unlikely to fully explain the contrasting findings for the younger and older age groups, although we cannot rule out some role.

Another explanation for the age contrast suggested here is the possibility that our prescriptions-based measure disproportionately overestimates PD onsets among under-50s, as these drugs may be used for other health conditions with overlapping symptoms, e.g. RLS and Dystonia. These conditions share some aspects of etiology with PD, involving dopaminergic dysfunction, and thus their treatment can include enhancing dopamine activity in the brain, like the treatment of PD. Indeed, comparing our own estimate of PD incidence with the figures implied by statistics from the National Institute for Health and Care Excellence (NICE)^45^ suggests that we may overcount PD cases in our dataset, and by a factor of around 1.5 (NICE suggested an incidence rate of 144 per 100,000 person-years in 2016). Based on that rate, we should expect approximately 2160 incidences of PD across our sample over 5 years, which is lower than the 3089 incidences we identified.) Additionally, considering a recent meta-analysis of gender-specific PD incidence^46^, the overcounting of PD onsets may be more pronounced in our younger age group. Therefore, a more cautious interpretation of our research is that it suggests a tentative association between exposure to air pollution and the onset of conditions—including, but not limited to, Parkinson’s disease (PD)—for which these drugs are prescribed in younger age groups. In support of this more cautious interpretation, sensitivity analysis using a stricter two-consecutive-period-prescription measure of PD onset returned non-significant estimated hazard ratios for both age groups. Given the limitations of our dataset, a more detailed investigation is beyond the scope of this paper. However, this is an important avenue for further research.

In addition to the potential measurement errors stemming from our prescriptions-based proxy for PD onset, this study has other limitations, including the inability to assess indoor pollution effects, despite growing evidence of the importance of indoor air pollution in health outcomes^47–49^, and reliance on modelled annual outdoor pollution averages. Furthermore, we could not examine short-term pollution events or exposures longer than 2 years, leaving the most appropriate exposure duration for modelling PD onset unclear. Finally, characterising the nature of the pollutants involved would add value to the study, since some components of particulate matter may have different effects on health than others^50,51^. Unfortunately, suitable speciation data do not exist for Northern Ireland. Despite these limitations, our findings contribute to the growing PD-pollution literature, highlighting potential but tentative associations at younger ages even in low-pollution contexts like Northern Ireland.

It is crucial to emphasise that our overall null finding should not undermine the importance of reducing population exposures to PM_2.5_ or NO_2_. Reducing these exposures remains vital due to robust evidence linking pollution to various health outcomes and emerging evidence of its association with PD in specific contexts^9,52–59^. Our data also tentatively suggest there may be an association with PD, or perhaps a broader class of conditions for which PD-related drugs are sometimes prescribed, among under-50s.

In conclusion, this study examines the link between medium-term air pollution exposure and PD onset using a large, nationally representative cohort from Northern Ireland. It benefits from complete address records which enable linkage to pollutant concentrations at the local level over an extended time frame, detailed Census data for statistical adjustment, and primary care prescription data, reducing the scope for the kinds of measurement errors associated with self-reports or hospital-based outcome measures^60–62^. The study also explores potential differences by sex and age. Overall, no significant associations were found between PM_2.5_, NO_2_ exposure, and PD onset for this cohort, or in males, females, and those aged 50+ years. However, we found some evidence of a statistically significant association between air pollution exposure and PD onset, using our prescriptions-based proxy, among under-50s. In the absence of alternative measures of PD onset for this cohort, it remains unclear how to interpret this estimated association. But it clearly warrants further research.

## Methods

### Data

We used data from a new linkage between the Northern Ireland Longitudinal Study (NILS), pollution data at the 1 km grid-square level, and the Enhanced Prescribing Database (EPD). The NILS is a longitudinal study that follows a 28% representative sample of the Northern Ireland population drawn from the NI Health Card Registration System, which contains address histories updated biannually. The NILS is linked to several other administrative datasets including Census records for 2011, which provided rich information on socioeconomic and demographic characteristics and contexts for sample members^63^. The pollution data, matched at the residential property level to NILS participants, provided annual 1 km grid-square modelled pollution data from 2009–2016 for both PM_2.5_ and NO_2_. These data were produced by Ricardo Energy & Environment for the UK Government’s air quality assessments^64^. These data were then linked to the EPD, which contains detailed information relating to all primary care prescriptions dispensed in Northern Ireland since March 2008^65^, made available to us at six-monthly frequency from January 2010 onwards. From this we extracted data on prescriptions in each 6-month period (January–June (hereafter semester 1) and July–December (hereafter semester 2)) of each year, from a defined list of items covering drugs that, according to the British National Formulary (BNF) classification system, were prescribed for PD at the time. (Note that, in the Northern Ireland health system the PD diagnostic process typically begins with a general practitioner (GP) conducting an initial assessment, followed by referral to a specialist—usually a neurologist or consultant with expertise in movement disorders—who is responsible for making the diagnosis.) All data were anonymized. Analysis was conducted in a trusted research environment under strict confidentiality and security protocols by ONS-accredited researchers. Researchers did not have access to addresses or other sensitive information.

### Analysis sample

Our main analysis sample—used for our preferred models—included all NILS members present in the 2011 Census who were aged 28 years or older at the time, had full address records, and were not in receipt of PD medication prior to the semester January–June 2012, which we treated as the first at-risk period for the purposes of modelling PD onset. The first at-risk period and associated sample exclusion condition was varied in sensitivity analysis. Compared to the full NILS sample returned in the 2011 Census and aged 28+ years at that time (but similarly excluding those with PD prescriptions prior to 2012 semester 1), our analysis sample was slightly (around 3.5%) smaller, mainly due to missing information on exposure to pollution at some point during the analysis period due to incomplete address records. In terms of measured characteristics, however, the two samples were very similar (see Table 1).

### Outcome variable

The outcome variable, drawn from the EPD, was set to 1 from the semester at which the individual had received any prescription for PD-related medication, including dopaminergic drugs, antimuscarinic drugs, and treatments for tremor, chorea, tics, and related disorders, and 0 otherwise (see Supplementary Table 23). Prescription data is commonly used in studies on air pollution and health^66–68^, but has rarely been used in the pollution and PD literature^19,33^.

Such prescription-based measures have advantages and disadvantages as proxies for the onset of PD. These measures are objective, requiring a prior PD diagnosis, but may undercount or overcount PD onsets. Undercounting is possible in cases where patients do not receive or delay receiving prescriptions, or where initial treatment occurs in hospitals rather than primary care. Such undercounting or delays could bias estimates if affected individuals were disproportionately exposed to higher or lower pollution levels. Sensitivity analysis varying exposure lag lengths was conducted to address these concerns, particularly relevant given evidence of longer diagnostic and prescription delays with increasing age of PD onset^43^. Alternatively, we may overcount PD onsets if prescriptions for drugs used to treat PD are sometimes issued to treat other conditions with overlapping symptoms, e.g. Restless Leg Syndrome (RLS) and Dystonia (For further information see NICE BNF online https://bnf.nice.org.uk/drug/ and https://bnfc.nice.org.uk/treatment-summaries/dystonias-and-related-disorders/#dopaminergic-drugs-used-in-dystonias, as well as, NHS website: https://www.nhs.uk/conditions/restless-legs-syndrome/treatment/). The direction of any resulting bias will depend on the extent to which these other conditions are themselves associated with air pollution exposure, which given a lack of existing evidence, remains unknown. Comparing the estimated incidence of PD using our prescriptions-based measure to that estimated by other methods suggested that overcounting was more likely an issue than undercounting, at least at younger ages. We assess this evidence and its implications for the interpretation of our estimates, particularly those where we split the sample by age group, in the discussion section.

### Exposure variables

Our exposure variables were derived from annual average PM_2.5_ and NO_2_ concentrations modelled at the 1 km grid-square level across Northern Ireland produced by Ricardo Energy & Environment for the UK Government’s air quality assessments. These concentrations were themselves derived from the aggregation of values from a variety of large and small point sources, as well as area and distance sources, using various datasets, including the UK National Atmospheric Emissions Inventory^64^. Modelled concentrations were calibrated with data from the UK national monitoring network and evaluated using data from monitoring sites not used in the calibration process prior to their publication. These data have been used in several existing studies of the health effects of ambient air pollution exposure within the UK^69,70^, including specifically in Northern Ireland^71^. Similar modelled pollution data have also been used extensively in the international literature^72–75^, including in the specific literature on the association between ambient air pollution and PD^16,32,34^. The trade-off for the population coverage that such data offer is the potential for measurement error in pollution concentrations. For an analysis of potential implications for estimated health effects of exposure see Samoli et al.^76^.

PM_2.5_ and NO_2_ are the two pollutants which have attracted most interest in the air pollution-PD literature to date, and our focus on these pollutants reflected this. All individuals in the sample were assigned exposure values for PM_2.5_ and NO_2_, via their residential address, for every 6-month period from January–June 2009 (2009 semester 1) through to July–December 2016 (2016 semester 2), although in our preferred models we use exposure data only from 2011 semester 1 onwards. Addresses are updated every 6 months in the NILS, in April and October of each year. We used April addresses to determine exposures for semester 1 and October addresses to determine exposures for semester 2 of each year. In other words, six-monthly exposures were assigned according to address at approximately the mid-point of each semester. Note that although the underlying pollution data were annual frequency, there was within-year variation in exposure in our analysis sample where (and only where) individuals changed residential address between April and October in any given year.

These data are best suited for studying medium-term exposures, initially defined as exposure over a 6-month period. Exposures are lagged by 1-year, reflecting evidence on the average delay between symptom onset and PD diagnosis^43^. Medium-term exposure durations in the literature range from weeks to years^77,78^. Sensitivity analysis included 2-year moving averages and varied lags for both 6-month and 2-year moving average exposures from zero (contemporaneous exposures) to 6 and 18 months.

### Covariates

The 2011 Census link allowed adjustment for a rich set of individual and household socio-economic and demographic covariates, each measured in March 2011. These variables are listed in Table 1, along with their (unweighted) sample means, both overall and separately for those in our analysis sample who did and did not experience PD onset. Approximately 0.1% of the sample had non-response (missing/edited) across multiple covariates, primarily from individuals in communal establishments during the 2011 Census, and were excluded from the sample. Table 1 also compares the analysis sample to the equivalent full sample of NILS members present in 2011 Census. We supplemented these covariates with neighbourhood deprivation indicators corresponding to 2011 Census residential address. These give the deprivation rank decile of the individual’s residential super output area (SOA) according to the 2010 multiple deprivation measure (MDM) index^79^. Note there are 890 SOAs in NI with an average population of 790 households.

### Model

We used time-dependent CPH models, with calendar time as the underlying time scale, to examine the associations between pollution exposures and the onset of PD, as is typical in existing cohort studies of air pollution and PD^4,19,30,34^. We estimated hazard ratios for the association between medium-term exposure to ambient PM_2.5_ and NO_2_ and receiving a first prescription for PD. Following Jo et al.^4^ exposure to ambient air pollution was modelled both in continuous/linear form and in categorical form (as quartiles) to allow for non-linearity. We estimated unadjusted models as well as models adjusted for covariates at individual, household and neighbourhood level. Note that 7.1% of individuals are right censored at some point during the analysis period due to death or emigration.

In our preferred model specification, our analysis of PD onset starts in the period 2012 semester 1 with exposures measured from the corresponding semester 1 year previously (2011 semester 1). This ensured that measured exposures did not precede measurement of the covariates. In sensitivity analysis we relaxed this restriction, modelling PD onsets from the first available data point (2010 semester 2) with corresponding exposures prior to the 2011 Census date, in addition to examining sensitivity to the length and lag of the exposure period. Standard errors were clustered at the SOA level. Analysis was performed in the trusted research environment of the Northern Ireland Statistics and Research Agency (NISRA) using STATA 17.

## Supplementary information

Supplementary information

## Data Availability

The datasets analysed in the current study are not publicly available per our data use agreement. The data, however, can be requested by accredited researchers from Northern Ireland Statistics and Research Agency (NISRA).
