# Supplementary material for: Exposure to ambient air pollution and onset of Parkinson’s disease in a large cohort study
Source: NPJ Parkinsons Dis. 2025 Oct 14;11:291. doi: 10.1038/s41531-025-01156-z (PMC12521372; doi:10.1038/s41531-025-01156-z)
Supplement: Supplementary file 1 — Supplementary information [file 41531_2025_1156_MOESM1_ESM.pdf]

Supplemental Content

Supplementary Figure 1. Hazard ratios for the association between medium-term exposure, with one year lag, to $PM_{2.5}$ and NO_2 and PD onset, overall sample, single and dual-pollutant models

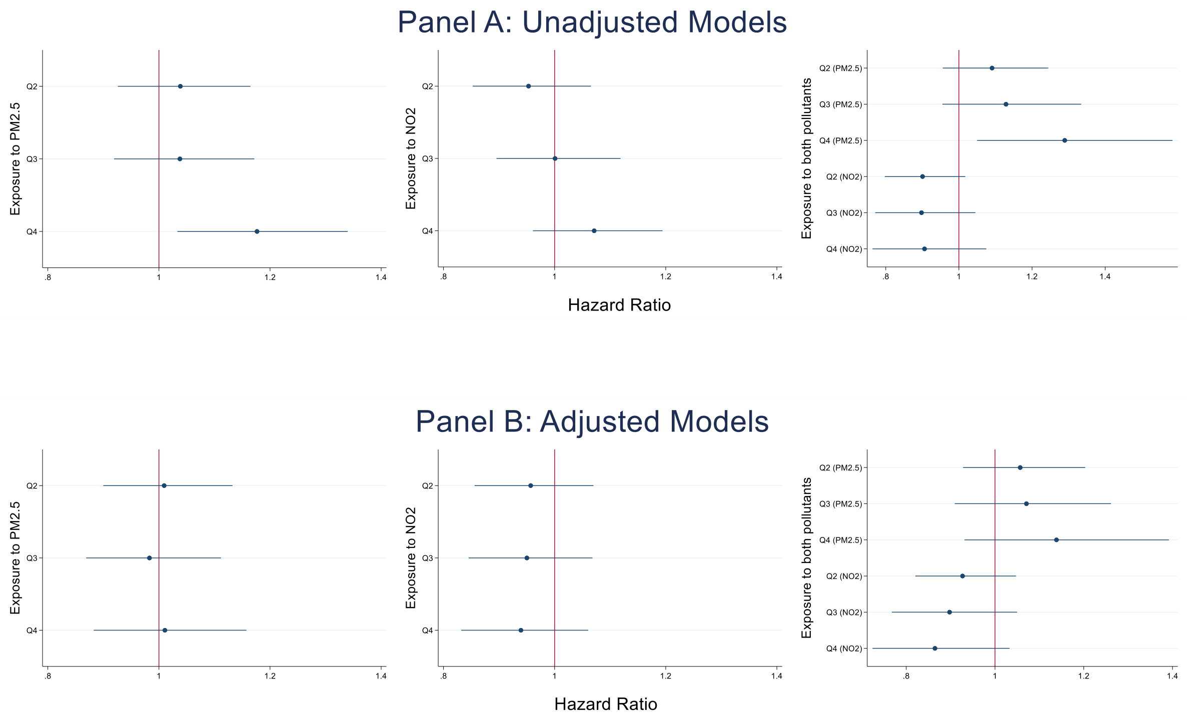

Note. All graphs show hazard ratios with 95% confidence intervals, based on coefficient estimates from a Cox Proportional Hazards (CPH) model with standard errors clustered by SOA. Panel A presents unadjusted model and Panel B presents adjusted model estimates controlling for covariates listed in Table 1. Hazard ratios for a 1 μg/m^3 increase in PM2.5 and NO2 are estimated using two separate models (the left-hand chart for PM2.5 and the middle chart for NO2) as well as a combined model including both pollutants (the right-hand chart). Only quartile models are presented.

Supplementary Table 1. Previous studies on the association between Parkinson Disease and $PM_{2.5}$ or NO_2 air pollution

Author (year of publication)	Country	Study design/Method	HR or OR (95% CI)	Mean exposure level	PD measure
**$PM_{2.5}$ (μg /m³)**					
Palacios et al. (2014)	USA	Cohort/CPH[1]	1.10 (0.83-1.45)	N/A	self-reported
Liu et al. (2016)	USA	Case-control/LR[2]	**1.29 (0.94-1.76)**	13.1[3]	Self-reported
Cerza et al. (2018)	Italy	Cohort/CPH	0.96 (0.92–1.01) per 5 μg/m3	17	Several sources Drug Prescriptions + Hospital discharges
Palacios et al. (2017)	USA	Cohort/CPH	0.97 (0.72-1.32)[4]	14.7[5]	Self-reported
Lee et al. (2017)[6]	South Korea	Case-control /LR	**1.61 (1.14-2.29)** per 10 μg/m3	N/A	emergency admission cases with primarily diagnosed PD
Shin et al. (2018)	Canada	Cohort/CPH	**1.04 (1.01-1.08)** for every IQR-increase	9.8	physician claims for PD
Salimi et al. (2020)	Australia	Cohort/LR	1.01 (0.98-1.04)	5.8	Self-reported
Shi et al. (2020)	USA	Cohort	**1.13 (1.12-1.14)** per 5 μg/m3	9.7	Hospital admission
Jo et al. (2021)	South Korea	Cohort/CPH	0.89 (0.60-1.32) HR for highest vs lowest quartile	26.5 (18.0-44.4)[7]	National Health Insurance registered Code

[1] Cox Proportional Hazard Models
[2] Logistic Regression
[3] Median of the 3rd quantile range
[4] Comparing the top to the bottom quintile of PM exposure
[5] Median of the 3rd quantile range
[6] This is an example for short term exposure effect following a unit increase in 8-day moving average.
[7] Median (range)

Wijngaarden et al. (2021)	USA	Cohort/Poisson	**1.06 (1.00, 1.12)** per interquartile range (IQR) increase	8.6-11.8 [8]	Hospital admission with relevant ICD codes
Rhew et al. (2021)	USA	Case-control/LR	1.13 (0.92-1.31)	10.27	Hospital admissions
Lee et al. (2022)	South Korea	Cohort /CPH	**1.19 (1.01-1.19)** per interquartile range (3.3 µg/m3)	30.4[9]	emergency admission cases with primarily diagnosed PD
Cole-Hunter et al. (2023)	Six European countries	Cohort/CPH	**1.25 (1.01-1.55)** per 5 µg/m3	N/A	PD mortality from mortality registries
Rumrich et al. (2023)	Finland	Case-Control/LR	0.99(0.96, 1.02) per interquartile range (3.9 µg/m3)	7.7	Incident PD diagnosis using Finnish special reimbursement register.
**NO_2 (ppm or µg /m^3)**					
Ritz et al. (2016)	Denmark	Case-control/LR	**1.21 (1.11-1.31)**	13.71 µg/m^3	National Hospital Register
Liu et al. (2016)	USA	Case-control/LR	1.02 (0.95-1.11)	11.8[10]	Self-reported
Cerza et al. (2016)	Italy	Cohort/CPH	0.97 (0.96–0.99) per 10 µg /m3 increase	42.7	
Lee et al. (2017)	South Korea	Case-control /LR	**2.35 (1.39-3.97)** per 10 ppb	N/A	emergency admission cases with primarily diagnosed PD
Shin et al. (2018)	Canada	Cohort/CPH	**1.03 (1.00-1.06)** for every IQR-increase	14.7 ppb	physician claims for PD
Salimi et al. (2020)	Australia	Cohort/LR	1.03 (0.98-1.08)	11.9 µg/m^3	Self-reported
Jo et al. (2021)	South Korea	Cohort/CPH	**1.41 (1.02-1.95)** HR for highest vs lowest quartile	0.033 (0.026-0.045) ppm[11]	National Health Insurance registered Code

[8] Range across various sites
[9] Median of the 3rd quantile range
[10] Median of the 3rd quantile range in ppb
[11] Median (range)

Cole-Hunter et al. (2023)	Six European countries	Cohort/CPH	1.13 (0.95–1.34) per 10 μg /m3 increase	N/A	PD mortality from mortality registries

Supplementary Table 2. Hazard ratios (95% CI) for the association between medium-term exposure to $PM_{2.5}$ and NO_2 and Parkinson's Disease onset, one year lag, overall sample, full regression results, Model 2

	$PM_{2.5}$		NO_2	
	Linear Model	Quartiles Model	Linear Model	Quartiles Model
**Lagged linear effect**	0.99		0.99	
	(0.96 - 1.02)		(0.98 - 1.00)	
**Lagged Quartiles effect**				
(Ref: 1st Quartile)				
Quartile 2		1.01		0.96
		(0.90 - 1.13)		(0.86 - 1.07)
Quartile 3		0.98		0.95
		(0.87 - 1.11)		(0.84 - 1.07)
Quartile 4		1.01		0.94
		(0.88 - 1.16)		(0.83 - 1.06)
Sex (Ref: Female)				
Male	0.82***	0.82***	0.82***	0.82***
	(0.76 - 0.89)	(0.76 - 0.89)	(0.76 - 0.89)	(0.76 - 0.89)
Age Group (Ref: < =30)				
31-35	1.41*	1.41*	1.41*	1.41*
	(1.02 – 1.97)	(1.02 - 1.97)	(1.02 – 1.97)	(1.02 - 1.97)
36-40	1.90***	1.91***	1.90***	1.90***
	(1.37 - 2.64)	(1.37 - 2.64)	(1.37 - 2.64)	(1.37 - 2.64)
41-45	1.95***	1.95***	1.95***	1.95***
	(1.42 - 2.68)	(1.42 - 2.68)	(1.42 - 2.67)	(1.42 - 2.67)
46-50	2.00***	2.00***	2.00***	2.00***
	(1.46 – 2.73)	(1.46 – 2.73)	(1.46 – 2.74)	(1.46 – 2.74)
51-55	2.17***	2.17***	2.17***	2.17***
	(1.59 - 2.97)	(1.59 - 2.97)	(1.59 - 2.97)	(1.59 - 2.97)
56-60	2.28***	2.28***	2.28***	2.28***
	(1.66 – 3.14)	(1.66 – 3.14)	(1.66 – 3.14)	(1.66 – 3.14)
61-65	2.77***	2.77***	2.77***	2.77***
	(2.00 – 3.82)	(2.00 – 3.82)	(2.00 – 3.82)	(2.00 – 3.82)
66-70	3.13***	3.13***	3.13***	3.13***
	(2.26 – 4.35)	(2.26 – 4.35)	(2.26 – 4.35)	(2.25 – 4.34)
71-75	3.73***	3.73***	3.73***	3.73***
	(2.68 – 5.18)	(2.68 – 5.18)	(2.69 – 5.18)	(2.68 – 5.17)
76-80	4.31***	4.31***	4.31***	4.30***
	(3.10 – 6.00)	(3.10 – 6.00)	(3.10 – 6.00)	(3.10 – 6.00)
80+	2.99***	2.99***	3.00***	2.99***
	(2.11 - 4.24)	(2.11 -4.24)	(2.11 - 4.25)	(2.11 -4.24)
COB (Ref: Northern Ireland)				
Rest of UK	0.99	0.99	0.98	0.99
	(0.84 - 1.16)	(0.84 - 1.16)	(0.83 - 1.16)	(0.84 - 1.16)
Republic of Ireland	0.87	0.87	0.86	0.86
	(0.69 - 1.09)	(0.69 - 1.09)	(0.68 - 1.08)	(0.69 - 1.09)
Born Elsewhere	0.65**	0.65**	0.65**	0.55**
	(0.48 - 0.89)	(0.48 - 0.89)	(0.48 - 0.89)	(0.48 - 0.89)
Education (Ref: No Qualifications)				
Below Degree or Equivalent	0.88*	0.88**	0.89*	0.88*
	(0.80 - 0.97)	(0.80 - 0.97)	(0.81 - 0.97)	(0.81 - 0.97)
Degree, Equivalent or above	0.74***	0.74***	0.75***	0.75***
	(0.66 - 0.83)	(0.66 - 0.83)	(0.67 - 0.83)	(0.67 - 0.83)

Economic Status (Ref: Inactive)				
Empl= Employed/self emp	0.72***	0.72***	0.73***	0.72***
	(0.65 – 0.81)	(0.65 – 0.81)	(0.65 – 0.81)	(0.65 – 0.81)
Marital Status (Ref: Never Married)				
Married	1.13	1.13	1.13	1.13
	(0.99 - 1.28)	(0.99 - 1.28)	(1.00 - 1.28)	(1.00 - 1.28)
Separated/Divorced/Widowed	1.08	1.08	1.08	1.08
	(0.95 - 1.23)	(0.95 - 1.23)	(0.94 - 1.23)	(0.95 - 1.24)
Other	0.61	0.61	0.60	0.60
	(0.19 - 1.89)	(0.19 - 1.89)	(0.19 - 1.89)	(0.19 - 1.89)
Religion (Ref: Catholic)				
Protestant or Other Christian/Religion	1.07	1.07	1.08	1.07
	(0.98 - 1.17)	(0.98 - 1.17)	(0.99 - 1.18)	(0.95 - 1.19)
No Religion or None Stated	1.06	1.06	1.07	1.07
	(0.94 - 1.21)	(0.94 – 1.21)	(0.95 - 1.22)	(0.94 - 1.21)
General Health (Ref: Bad/Very bad Health)				
health = 1, Fair/Good/Very good GH	0.72***	0.72***	0.72***	0.72***
	(0.657 - 0.80)	(0.65 - 0.80)	(0.65 - 0.80)	(0.65 - 0.80)
LTAL Illness (Ref: NO)				
With Long-Term Illness	1.99***	1.99***	1.91***	1.91***
	(1.81 – 2.18)	(1.81 – 2.18)	(1.72 – 2.12)	(1.73– 2.12)
No. of Children in Household (Ref: 0)				
1	0.90	0.95	0.90	0.99
	(0.78 - 1.03)	(0.78 - 1.03)	(0.78 - 1.04)	(0.78 - 1.04)
2	0.74***	0.74***	0.74***	0.74***
	(0.63 - 0.88)	(0.63 - 0.88)	(0.63 - 0.83)	(0.63 - 0.83)
3+	0.90	0.90	0.89	0.89
	(0.73 - 1.10)	(0.73 - 1.10)	(0.72 - 1.10)	(0.72 - 1.10)
No. of Cars in Household (Ref: 0)				
1	0.99	1.00	0.99	0.99
	(0.90 - 1.10)	(0.90- 1.11)	(0.89 - 1.09)	(0.89 - 1.10)
2	0.90	0.91	0.89	0.90
	(0.79 -1.03)	(0.80 – 1.04)	(0.78 - 1.02)	(0.78 - 1.02)
3+	0.96	0.97	0.94	0.95
	(0.82 - 1.14)	(0.82 - 1.15)	(0.80 - 1.11)	(0.80 - 1.13)
People per room (Ref: >1)				
ppr = 1	1.01	1.01	1.01	1.01
	(0.73 - 1.40)	(0.73 - 1.40)	(0.73 - 1.40)	(0.73 - 1.40)
2010 Multiple Deprivation Measure (Ref: 1 (Most deprived))				
2	1.01	1.02	0.98	1.01
	(0.86 - 1.19)	(0.87 - 1.20)	(0.84 - 1.16)	(0.86 - 1.19)
3	1.03	1.04	1.00	1.03
	(0.88 - 1.21)	(0.89 - 1.22)	(0.85 - 1.18)	(0.87 - 1.20)
4	1.08	1.09	1.03	1.06
	(0.91 - 1.27)	(0.93 - 1.28)	(0.87 - 1.21)	(0.90 - 1.25)
5	1.06	1.07	1.01	1.04
	(0.88 - 1.26)	(0.90 - 1.27)	(0.85 - 1.21)	(0.87 - 1.24)
6	0.89	0.90	0.85	0.87
	(0.74 - 1.06)	(0.76 – 1.07)	(0.71 - 1.01)	(0.73 – 1.04)
7	0.96	0.97	0.92	0.95
	(0.80 - 1.15)	(0.81 - 1.16)	(0.76 - 1.11)	(0.79 - 1.14)
8	1.01	1.02	0.97	1.00

	(0.85 - 1.19)	(0.86 - 1.21)	(0.81 - 1.15)	(0.84 - 1.19)
9	0.93	0.93	0.90	0.92
	(0.78 - 1.10)	(0.79 - 1.11)	(0.75 - 1.07)	(0.78 - 1.10)
10 (Least deprived)	0.88	0.89	0.86	0.89
	(0.72 - 1.08)	(0.73 - 1.09)	(0.71 - 1.05)	(0.73 - 1.08)
Observations	2688153	2688153	2688153	2688153

*Notes. Each model is a Cox Proportional Hazard (CPH) model with standard error clustered by SOA. Each cell presents the estimated hazard ratio for a 1 μg/m^3 increase in $PM_{2.5}$ and NO_2 along with the 95% confidence interval in parentheses. The table reports a medium-term exposure effect defined as exposure to pollution over the semester with one year lag. Covariates are at individual, household and neighbourhood levels listed in Table 1. * $p < 0.05$, ** $p < 0.01$, *** $p < 0.001$.*

Supplementary Table 3. Hazard ratios (95% CI) for the association between medium-term exposure to $PM_{2.5}$ and NO_2 and Parkinson's disease, one year lag, extended sample

	Model 1	Model 2
Panel A ($PM_{2.5}$, μg/m^3)		
Linear model	1.05* (1.01-1.09)	1.00 (0.95-1.04)
Quartiles model		
1st (reference)		
2nd	1.03 (0.91-1.15)	0.99 (0.88-1.12)
3rd	1.01 (0.90-1.14)	0.95 (0.84-1.08)
4th	1.16* (1.02-1.32)	0.99 (0.87-1.14)
Observations	2765090	2765090
Covariates	NO	YES
Panel B (NO_2, μg/m^3)		
Linear model	1.03 (0.99-1.06)	0.98 (0.94-1.02)
Quartiles model		
1st (reference)		
2nd	0.96 (0.86-1.07)	0.96 (0.86-1.07)
3rd	1.00 (0.89-1.11)	0.94 (0.84-1.06)
4th	1.08 (0.97-1.21)	0.94 (0.83-1.06)
Observations	2762062	2762062
Covariates	NO	YES

*Notes. Each model is a Cox Proportional Hazard (CPH) model with standard error clustered by SOA. Each cell presents the estimated hazard ratio for a 1 μg/m^3 increase in $PM_{2.5}$ (Panel A) and NO_2 (Panel B) along with the 95% confidence interval in parentheses. The extended sample includes those excluded from the original analysis sample on grounds of missing exposure values, with missing values imputed using exposure information in adjacent periods. The table reports a medium-term exposure effect defined as exposure to pollution over the semester with one year lag. Covariates are at individual, household and neighbourhood levels listed in Table 1. * $p < 0.05$, ** $p < 0.01$, *** $p < 0.001$.*

Supplementary Table 4. Hazard ratios (95% CI) for the association between medium-term exposure to $PM_{2.5}$ and NO_2 and Parkinson's disease, overall sample, one year lag, extended at-risk period

	Model 1	Model 2
Panel A ($PM_{2.5}$, μg/m³)		
Linear model	1.04*<br>(1.01-1.06)	0.99<br>(0.97-1.02)
Quartiles model		
1st (reference)		
2nd	1.06<br>(0.95-1.18)	1.03<br>(0.92-1.15)
3rd	1.03<br>(0.92-1.16)	0.97<br>(0.86-1.10)
4th	1.17*<br>(1.03-1.32)	1.00<br>(0.87-1.13)
Observations	3234774	3234774
Covariates	NO	YES
Panel B (NO_2, μg/m³)		
Linear model	1.01<br>(1.00-1.01)	0.99<br>(0.98-1.00)
Quartiles model		
1st (reference)		
2nd	1.00<br>(0.91-1.11)	1.00<br>(0.90-1.11)
3rd	1.01<br>(0.91-1.13)	0.96<br>(0.86-1.07)
4th	1.07<br>(0.97-1.19)	0.93<br>(0.83-1.04)
Observations	3234774	3234774
Covariates	NO	YES

*Notes. Each model is a Cox Proportional Hazard (CPH) model with standard error clustered by SOA. Each cell presents the estimated hazard ratio for a 1 μg/m³ increase in $PM_{2.5}$ (Panel A) and NO_2 (Panel B) along with the 95% confidence interval in parentheses. The extended at-risk period runs from 2010 S2. The table reports a medium-term exposure effect defined as exposure to pollution over the semester with one year lag. Covariates are at individual, household and neighbourhood levels listed in Table 1. * $p < 0.05$, ** $p < 0.01$, *** $p < 0.001$.*

Supplementary Table 5. Hazard ratios (95% CI) for the association between medium-term exposure to $PM_{2.5}$ and NO_2 and Parkinson's disease, overall sample, contemporaneous exposures

	Model 1	Model 2
Panel A ($PM_{2.5}$, μg/m³)		
Linear model	1.02* (1.00-1.05)	0.98 (0.96-1.01)
Quartiles model		
1st (reference)		
2nd	1.10 (0.99-1.22)	1.06 (0.95-1.18)
3rd	1.03 (0.91-1.15)	0.96 (0.86-1.09)
4th	1.15* (1.02-1.29)	0.97 (0.86-1.11)
Observations	2697318	2697318
Covariates	NO	YES
Panel B (NO_2, μg/m³)		
Linear model	1.00 (1.00-1.01)	0.99* (0.98-1.00)
Quartiles model		
1st (reference)		
2nd	1.04 (0.93-1.16)	1.04 (0.93-1.16)
3rd	1.04 (0.93-1.16)	0.99 (0.88-1.11)
4th	1.08 (0.97-1.21)	0.95 (0.84-1.08)
Observations	2697318	2697318
Covariates	NO	YES

*Notes. Each model is a Cox Proportional Hazard (CPH) model with standard error clustered by SOA. Each cell presents the estimated hazard ratio for a 1 μg/m³ increase in $PM_{2.5}$ (Panel A) and NO_2 (Panel B) along with the 95% confidence interval in parentheses. The table reports a medium-term exposure effect defined as exposure to pollution over the semester with no lag. Covariates are at individual, household and neighbourhood levels listed in Table 1. * $p < 0.05$, ** $p < 0.01$, *** $p < 0.001$.*

Supplementary Table 6. Hazard ratios (95% CI) for the association between medium-term exposure to $PM_{2.5}$ and NO_2 and Parkinson's disease, overall sample, 6 months lag exposures

	Model 1	Model 2
Panel A ($PM_{2.5}$, μg/m^3)		
Linear model	1.03* (1.00-1.06)	0.99 (0.96-1.02)
Quartiles model		
1st (reference)		
2nd	1.07 (0.96-1.19)	1.03 (0.92-1.15)
3rd	1.01 (0.89-1.13)	0.95 (0.84-1.07)
4th	1.17* (1.03-1.32)	1.00 (0.88-1.14)
Observations	2693667	2693667
Covariates	NO	YES
Panel B (NO_2, μg/m^3)		
Linear model	1.00 (1.00-1.01)	0.99 (0.98-1.00)
Quartiles model		
1st (reference)		
2nd	0.98 (0.88-1.10)	0.99 (0.89-1.10)
3rd	1.02 (0.92-1.14)	0.97 (0.87-1.09)
4th	1.11 (0.99-1.23)	0.98 (0.87-1.11)
Observations	2693667	2693667
Covariates	NO	YES

*Notes. Each model is a Cox Proportional Hazard (CPH) model with standard error clustered by SOA. Each cell presents the estimated hazard ratio for a 1 μg/m^3 increase in $PM_{2.5}$ (Panel A) and NO_2 (Panel B) along with the 95% confidence interval in parentheses. The table reports a medium-term exposure effect defined as exposure to pollution over the semester with 6 months lag. Covariates are at individual, household and neighbourhood levels listed in Table 1. * $p < 0.05$, ** $p < 0.01$, *** $p < 0.001$.*

Supplementary Table 7. Hazard ratios (95% CI) for the association between medium-term exposure to $PM_{2.5}$ and NO_2 and Parkinson's disease, overall sample, 18 months lag exposures

	Model 1	Model 2
Panel A ($PM_{2.5}$, µg/m^3)		
Linear model	1.03*<br>(1.00-1.06)	0.99<br>(0.96-1.02)
Quartiles model		
1st (reference)		
2nd	1.01<br>(0.88-1.14)	1.00<br>(0.88-1.14)
3rd	1.05<br>(0.92-1.19)	1.01<br>(0.89-1.15)
4th	1.11<br>(0.97-1.27)	0.97<br>(0.84-1.12)
Observations	2681410	2681410
Covariates	NO	YES
Panel B (NO_2, µg/m^3)		
Linear model	1.00<br>(1.00-1.01)	0.99<br>(0.98-1.00)
Quartiles model		
1st (reference)		
2nd	1.02<br>(0.91-1.14)	1.03<br>(0.92-1.15)
3rd	1.03<br>(0.92-1.15)	0.98<br>(0.87-1.10)
4th	1.07<br>(0.96-1.20)	0.94<br>(0.83-1.06)
Observations	2681410	2681410
Covariates	NO	YES

*Notes. Each model is a Cox Proportional Hazard (CPH) model with standard error clustered by SOA. Each cell presents the estimated hazard ratio for a 1 µg/m^3 increase in $PM_{2.5}$ (Panel A) and NO_2 (Panel B) along with the 95% confidence interval in parentheses. The table reports a medium-term exposure effect defined as exposure to pollution over the semester with 18 months lag. Covariates are at individual, household and neighbourhood levels listed in Table 1. * $p < 0.05$, ** $p < 0.01$, *** $p < 0.001$.*

Supplementary Table 8. Hazard ratios (95% CI) for the association between medium-term exposure to $PM_{2.5}$ and NO_2 and Parkinson's disease, overall sample, 18 months lag exposures with first at-risk-period moved to 2012 S2

	Model 1	Model 2
Panel A ($PM_{2.5}$, μg/m³)		
Linear model	1.04** (1.01-1.07)	1.00 (0.97-1.03)
Quartiles model		
1st (reference)		
2nd	1.02 (0.90-1.17)	1.02 (0.89-1.16)
3rd	1.11 (0.98-1.27)	1.08 (0.94-1.23)
4th	1.16* (1.01-1.33)	1.01 (0.87-1.17)
Observations	2408140	2408140
Covariates	NO	YES
Panel B (NO_2, μg/m³)		
Linear model	1.01 (1.00-1.01)	0.99 (0.98-1.00)
Quartiles model		
1st (reference)		
2nd	1.02 (0.91-1.15)	1.03 (0.92-1.16)
3rd	1.06 (0.95-1.19)	1.01 (0.90-1.14)
4th	1.09 (0.97-1.22)	0.96 (0.84-1.09)
Observations	2408140	2408140
Covariates	NO	YES

*Notes. Each model is a Cox Proportional Hazard (CPH) model with standard error clustered by SOA. Each cell presents the estimated hazard ratio for a 1 μg/m³ increase in $PM_{2.5}$ (Panel A) and NO_2 (Panel B) along with the 95% confidence interval in parentheses. The table reports a medium-term exposure effect defined as exposure to pollution over the semester with 18 months lag where first at-risk period is 2012 S2. Covariates are at individual, household and neighbourhood levels listed in Table 1. * $p < 0.05$, ** $p < 0.01$, *** $p < 0.001$.*

Supplementary Table 9. Hazard ratios (95% CI) for the association between medium-term exposure to $PM_{2.5}$ and NO_2 and Parkinson's disease, overall sample, 24 months lag exposures with first at-risk-period moved to 2013 S1

	Model 1	Model 2
Panel A ($PM_{2.5}$, μg/m^3)		
Linear model	1.03*<br>(1.01-1.06)	0.99<br>(0.96-1.02)
Quartiles model		
1st (reference)		
2nd	1.01<br>(0.87-1.17)	1.03<br>(0.89-1.20)
3rd	1.07<br>(0.92-1.23)	1.06<br>(0.90-1.23)
4th	1.12<br>(0.97-1.29)	0.99<br>(0.85-1.16)
Observations	2130285	2130285
Covariates	NO	YES
Panel B (NO_2, μg/m^3)		
Linear model	1.01<br>(1.00-1.02)	0.99<br>(0.98-1.00)
Quartiles model		
1st (reference)		
2nd	1.09<br>(0.97-1.24)	1.11<br>(0.98-1.26)
3rd	1.08<br>(0.95-1.22)	1.04<br>(0.92-1.19)
4th	1.14*<br>(1.01-1.29)	1.01<br>(0.88-1.16)
Observations	2130285	2130285
Covariates	NO	YES

*Notes. Each model is a Cox Proportional Hazard (CPH) model with standard error clustered by SOA. Each cell presents the estimated hazard ratio for a 1 μg/m^3 increase in $PM_{2.5}$ (Panel A) and NO_2 (Panel B) along with the 95% confidence interval in parentheses. The table reports a medium-term exposure effect defined as exposure to pollution over the semester with 18 months lag where first at-risk period is 2013 S1. Covariates are at individual, household and neighbourhood levels listed in Table 1. * $p < 0.05$, ** $p < 0.01$, *** $p < 0.001$.*

Supplementary Table 10. Hazard ratios (95% CI) for the association between 2-year moving average exposure to $PM_{2.5}$ and NO_2 and Parkinson's disease, overall sample with one year lag

	Model 1	Model 2
Panel A ($PM_{2.5}$, μg/m³)		
Linear model	1.03<br>(1.00-1.05)	0.98<br>(0.95-1.01)
Quartiles model		
1st (reference)		
2nd	0.96<br>(0.85-1.08)	0.98<br>(0.86-1.11)
3rd	1.03<br>(0.91-1.16)	1.01<br>(0.89-1.14)
4th	1.08<br>(0.96-1.21)	0.95<br>(0.84-1.09)
Observations	2639622	2639622
Covariates	NO	YES
Panel B (NO_2, μg/m³)		
Linear model	1.00<br>(1.00-1.01)	0.99<br>(0.98-1.00)
Quartiles model		
1st (reference)		
2nd	1.00<br>(0.89-1.12)	1.01<br>(0.90-1.14)
3rd	0.99<br>(0.88-1.11)	0.95<br>(0.84-1.07)
4th	1.09<br>(0.97-1.21)	0.96<br>(0.85-1.09)
Observations	2639608	2639608
Covariates	NO	YES

*Notes. Each model is a Cox Proportional Hazard (CPH) model with standard error clustered by SOA. Each cell presents the estimated hazard ratio for a 1 μg/m³ increase in $PM_{2.5}$ (Panel A) and NO_2 (Panel B) along with the 95% confidence interval in parentheses. The table reports 2-year moving average exposure effect lagged one year. Covariates are at individual, household and neighbourhood levels listed in Table 1. * $p < 0.05$, ** $p < 0.01$, *** $p < 0.001$.*

Supplementary Table 11. Hazard ratios (95% CI) for the association between 2-year moving average exposure to $PM_{2.5}$ and NO_2 and Parkinson's disease, overall sample with 18 months lag

	Model 1	Model 2
Panel A ($PM_{2.5}$, μg/m^3)		
Linear model	1.02<br>(0.99-1.05)	0.98<br>(0.95-1.01)
Quartiles model		
1st (reference)		
2nd	0.89*<br>(0.79-0.99)	0.90<br>(0.80-1.01)
3rd	0.99<br>(0.89-1.12)	0.97<br>(0.86-1.09)
4th	1.03<br>(0.92-1.15)	0.91<br>(0.81-1.03)
Observations	2632661	2632661
Covariates	NO	YES
Panel B (NO_2, μg/m^3)		
Linear model	1.00<br>(1.00-1.01)	0.99<br>(0.98-1.00)
Quartiles model		
1st (reference)		
2nd	0.99<br>(0.88-1.11)	1.01<br>(0.90-1.13)
3rd	1.00<br>(0.89-1.12)	0.97<br>(0.86-1.10)
4th	1.11<br>(0.99-1.24)	0.99<br>(0.88-1.12)
Observations	2632644	2632644
Covariates	NO	YES

*Notes. Each model is a Cox Proportional Hazard (CPH) model with standard error clustered by SOA. Each cell presents the estimated hazard ratio for a 1 μg/m^3 increase in $PM_{2.5}$ (Panel A) and NO_2 (Panel B) along with the 95% confidence interval in parentheses. The table reports 2-year moving average exposure effect lagged 18 months. Covariates are at individual, household and neighbourhood levels listed in Table 1. * $p < 0.05$, ** $p < 0.01$, *** $p < 0.001$.*

Supplementary Table 12. Hazard ratios (95% CI) for the association between 2-year moving average exposure to $PM_{2.5}$ and NO_2 and Parkinson's disease, overall sample, contemporaneous exposure

	Model 1	Model 2
Panel A ($PM_{2.5}$, μg/m³)		
Linear model	1.03*<br>(1.00-1.06)	0.99<br>(0.96-1.02)
Quartiles model		
1st (reference)		
2nd	0.97<br>(0.87-1.08)	0.98<br>(0.87-1.09)
3rd	0.99<br>(0.89-1.11)	0.95<br>(0.84-1.07)
4th	1.10<br>(0.98-1.24)	0.96<br>(0.85-1.09)
Observations	2649926	2649926
Covariates	NO	YES
Panel B (NO_2, μg/m³)		
Linear model	1.00<br>(1.00-1.01)	0.99<br>(0.98-1.00)
Quartiles model		
1st (reference)		
2nd	1.04<br>(0.93-1.17)	1.06<br>(0.95-1.19)
3rd	1.03<br>(0.92-1.16)	1.00<br>(0.89-1.13)
4th	1.11<br>(0.99-1.24)	0.99<br>(0.88-1.12)
Observations	2649917	2649917
Covariates	NO	YES

*Notes. Each model is a Cox Proportional Hazard (CPH) model with standard error clustered by SOA. Each cell presents the estimated hazard ratio for a 1 μg/m³ increase in $PM_{2.5}$ (Panel A) and NO_2 (Panel B) along with the 95% confidence interval in parentheses. The table reports 2-year moving average exposure effect with no lag. Covariates are at individual, household and neighbourhood levels listed in Table 1. * $p < 0.05$, ** $p < 0.01$, *** $p < 0.001$.*

Supplementary Table 13. Hazard ratios (95% CI) for the association between medium-term exposure to $PM_{2.5}$ and NO_2 and Parkinson's Disease onset, one year lag, overall sample with alternative definition of PD

	Model 1	Model 2
Panel A ($PM_{2.5}$, μg/m³)		
Linear model	1.03<br>(0.99-1.07)	0.98<br>(0.94-1.02)
Quartiles model		
1st (reference)		
2nd	1.04<br>(0.88-1.23)	1.02<br>(0.86-1.21)
3rd	1.04<br>(0.88-1.23)	0.99<br>(0.83-1.17)
4th	1.16<br>(0.98-1.38)	0.99<br>(0.83-1.19)
Observations	2695756	2695756
Covariates	NO	YES
Panel B (NO_2, μg/m³)		
Linear model	1.01<br>(1.00-1.02)	0.99<br>(0.98-1.00)
Quartiles model		
1st (reference)		
2nd	0.89<br>(0.77-1.02)	0.89<br>(0.77-1.03)
3rd	1.02<br>(0.88-1.18)	0.97<br>(0.83-1.14)
4th	1.06<br>(0.92-1.23)	0.92<br>(0.78-1.08)
Observations	2695756	2695756
Covariates	NO	YES

*Notes. Each model is a Cox Proportional Hazard (CPH) model with standard error clustered by SOA. Each cell presents the estimated hazard ratio for a 1 μg/m3 increase in PM2.5 (Panel A) and NO2 (Panel B) along with the 95% confidence interval in parentheses. Parkinson disease defines as subjects receiving at least two prescriptions in two consecutive semesters. The table reports a medium-term exposure effect defined as exposure to pollution over the semester with one year lag. Covariates are at individual, household and neighbourhood levels listed in Table 1. * p < 0.05, ** p < 0.01, *** p <0.001.*

Supplementary Table 14. Estimated hazard ratios (95% CI) for the association between medium-term exposure to $PM_{2.5}$ and NO_2 and Parkinson's Disease onset, one year lag, overall sample, comparison of Table 2 estimates with estimates from Cox model stratified by age, sex and education

	Model 2 (from Table 2)	Model 2 (stratified)
Panel A ($PM_{2.5}$, μg/m^3)		
Linear model	0.99 (0.96-1.02)	0.99 (0.96-1.02)
Quartiles model		
1st (reference)		
2nd	1.01 (0.90-1.13)	1.00 (0.89-1.12)
3rd	0.98 (0.87-1.11)	0.97 (0.86-1.10)
4th	1.01 (0.88-1.16)	1.00 (0.87-1.15)
Observations	2688153	2688153
Covariates	YES	YES
Panel B (NO_2, μg/m^3)		
Linear model	0.99 (0.98-1.00)	0.95 (0.85-1.06)
Quartiles model		
1st (reference)		
2nd	0.96 (0.86-1.07)	0.95 (0.85-1.06)
3rd	0.95 (0.84-1.07)	0.94 (0.84-1.06)
4th	0.94 (0.83-1.06)	0.94 (0.83-1.07)
Observations	2688153	2688153
Covariates	YES	YES

Notes. Model 2 (from Table 2) is the original adjusted model in the paper (Table 2) where we control for all listed covariates. Model 2 (stratified) is the otherwise equivalent model but stratified by age, sex and education.

Supplementary Table 15. Estimated hazard ratios (95% CI) for the association between medium-term exposure to $PM_{2.5}$ and NO_2 and Parkinson's Disease onset, one year lag, overall sample without controls for health (GH and LTI Variables)

	Model 2 (from Table 2)	Model 2 (without health variable)
Panel A ($PM_{2.5}$, μg/m^3)		
Linear model	0.99 (0.96-1.02)	1.00 (0.97-1.03)
Quartiles model		
1st (reference)		
2nd	1.01 (0.90-1.13)	1.01 (0.90-1.13)
3rd	0.98 (0.87-1.11)	0.98 (0.87-1.11)
4th	1.01 (0.88-1.16)	1.02 (0.89-1.17)
Observations	2688153	2688153
Covariates	YES	YES
Panel B (NO_2, μg/m^3)		
Linear model	0.99 (0.98-1.00)	0.99 (0.98-1.00)
Quartiles model		
1st (reference)		
2nd	0.96 (0.86-1.07)	0.95 (0.85-1.06)
3rd	0.95 (0.84-1.07)	0.95 (0.84-1.07)
4th	0.94 (0.83-1.06)	0.95 (0.84-1.07)
Observations	2688153	2688153
Covariates	YES	YES

*Notes. Each model is a Cox Proportional Hazard (CPH) model with standard error clustered by SOA. Each cell presents the estimated hazard ratio for a 1 μg/m3 increase in PM2.5 (Panel A) and NO2 (Panel B) along with the 95% confidence interval in parentheses. The table reports a medium-term exposure effect defined as exposure to pollution over the semester with one year lag. Covariates are at individual, household and neighbourhood levels as listed in Table 1. * p < 0.05, ** p < 0.01, *** p <0.001*

Supplementary Table 16. Estimated hazard ratios (95% CI) for the association between medium-term exposure to $PM_{2.5}$ and NO_2 and Parkinson's Disease onset, one year lag, overall sample with additional controls for health

	Model 2 (+Diab & CVD)		Model 2 (+Chronic Illness)	
	(a) linear	(b) quartiles	(a) linear	(b) quartiles
Panel A ($PM_{2.5}$, μg/m³)				
Linear model	0.99<br>(0.96-1.02)		0.99<br>(0.96-1.02)	
Quartiles model				
1st (reference)				
2nd		1.01<br>(0.90-1.13)		1.01<br>(0.90-1.13)
3rd		0.98<br>(0.87-1.11)		0.98<br>(0.87-1.11)
4th		1.01<br>(0.88-116)		1.01<br>(0.88-116)
Cardio	1.64***<br>(1.48-1.81)	1.64***<br>(1.48-1.81)		
Diabetes	0.90<br>(0.79-1.01)	0.90<br>(0.79-1.01)		
Chronic Illness			0.99<br>(0.89-1.10)	0.99<br>(0.89-1.10)
Observations	2688153	2688153	2688153	2688153
Panel B (NO_2, μg/m³)				
Linear model	0.99<br>(0.98-1.00)		0.99<br>(0.98-1.00)	
Quartiles model				
1st (reference)				
2nd		0.96<br>(0.86-1.07)		0.96<br>(0.86-1.07)
3rd		0.95<br>(0.84-1.07)		0.95<br>(0.84-1.07)
4th		0.94<br>(0.83-1.06)		0.94<br>(0.83-1.06)
Cardio	1.64***<br>(1.48-1.81)	1.64***<br>(1.48-1.81)		
Diabetes	0.90<br>(0.79-1.01)	0.90<br>(0.79-1.01)		
Chronic Illness			0.99<br>(0.89-1.10)	0.99<br>(0.89-1.10)
Observations	2688153	2688153	2688153	2688153

*Notes. Each model is a Cox Proportional Hazard (CPH) model with standard error clustered by SOA. Each cell presents the estimated hazard ratio for a 1 μg/m3 increase in PM2.5 (Panel A) and NO2 (Panel B) along with the 95% confidence interval in parentheses. The table reports a medium-term exposure effect defined as exposure to pollution over the semester with one year lag. Covariates are at individual, household and neighbourhood levels as listed in Table 1. * $p < 0.05$, ** $p < 0.01$, *** $p < 0.001$*

Supplementary Table 17. Hazard ratios (95% CI) for the association between medium-term exposure to $PM_{2.5}$ and NO_2 and Parkinson's disease, overall sample, contemporaneous exposure, by age

	Age in 2011 < 50 years		Age in 2011 >= 50 years	
	Model 1	Model 2	Model 1	Model 2
Panel A ($PM_{2.5}$, μg/m³)				
Linear model	1.10***	1.03	0.99	0.97
	(1.05 -1.14)	(0.98 -1.08)	(0.96-1.02)	(0.94-1.00)
Quartiles model				
1st (reference)				
2nd	1.22*	1.15	1.04	1.02
	(1.01-1.49)	(0.94-1.40)	(0.92-1.18)	(0.90-1.16)
3rd	1.21	1.10	0.94	0.92
	(0.98-1.49)	(0.88-1.37)	(0.82-1.08)	(0.79-1.06)
4th	1.50***	1.16	1.01	0.92
	(1.21-1.85)	(0.92-1.46)	(0.88-1.17)	(0.79-1.07)
Observations	1419326	1419326	1277992	1277992
Covariates	NO	YES	NO	YES
Panel B (NO_2, μg/m³)				
Linear model	1.02***	1.00	0.99	0.99*
	(1.01-1.04)	(0.99-1.02)	(0.99-1.01)	(0.98-1.00)
Quartiles model				
1st (reference)				
2nd	0.94	0.94	1.07	1.07
	(0.77-1.15)	(0.76-1.15)	(0.95-1.21)	(0.95-1.22)
3rd	1.32**	1.26*	0.93	0.90
	(1.09-1.61)	(1.02-1.56)	(0.81-1.05)	(0.79-1.03)
4th	1.35***	1.14	0.98	0.89
	(1.10-1.65)	(0.91-1.44)	(0.85-1.11)	(0.77-1.03)
Observations	1419326	1419326	1277992	1277992
Covariates	NO	YES	NO	YES

*Notes. Each model is a Cox Proportional Hazard (CPH) model with standard error clustered by SOA. Sample splits by age below and above 50, and each cell presents the estimated hazard ratio for a 1 μg/m3 increase in PM2.5 (Panel A) and NO2 (Panel B) along with the 95% confidence interval in parentheses. The table reports a medium-term exposure effect defined as exposure to pollution over the semester with no lag. Covariates are at individual, household and neighbourhood levels listed in Table 1. * p < 0.05, ** p < 0.01, *** p <0.001.*

Supplementary Table 18. Hazard ratios (95% CI) for the association between medium-term exposure to $PM_{2.5}$ and NO_2 and Parkinson's disease, overall sample with 6 months lag, by age

	Age in 2011 < 50 years		Age in 2011 >= 50 years	
	Model 1	Model 2	Model 1	Model 2
Panel A ($PM_{2.5}$, μg/m³)				
Linear model	1.10***	1.03	1.00	0.98
	(1.05 -1.15)	(0.98 -1.08)	(0.97-1.03)	(0.94-1.01)
Quartiles model				
1st (reference)				
2nd	1.28*	1.20	0.98	0.97
	(1.05-1.56)	(0.98-1.47)	(0.86-1.12)	(0.86-1.10)
3rd	1.17	1.05	0.93	0.91
	(0.93-1.45)	(0.84-1.33)	(0.81-1.07)	(0.79-1.05)
4th	1.50***	1.15	1.04	0.95
	(1.20-1.87)	(0.91-1.47)	(0.90-1.20)	(0.82-1.11)
Observations	1416956	1416956	1276711	1276711
Covariates	NO	YES	NO	YES
Panel B (NO_2, μg/m³)				
Linear model	1.02***	1.00	1.00	0.99*
	(1.01-1.04)	(0.99-1.02)	(0.99-1.01)	(0.98-1.00)
Quartiles model				
1st (reference)				
2nd	0.95	0.95	0.99	1.00
	(0.77-1.16)	(0.77-1.17)	(0.88-1.13)	(0.88-1.13)
3rd	1.32**	1.26*	0.90	0.88
	(1.08-1.61)	(1.02-1.56)	(0.80-1.02)	(0.77-1.00)
4th	1.43***	1.23	0.98	0.91
	(1.17-1.75)	(0.98-1.55)	(0.87-1.12)	(0.79-1.05)
Observations	1416956	1416956	1276711	1276711
Controls	NO	YES	NO	YES

*Notes. Each model is a Cox Proportional Hazard (CPH) model with standard error clustered by SOA. Sample splits by age below and above 50, and each cell presents the estimated hazard ratio for a 1 μg/m3 increase in PM2.5 (Panel A) and NO2 (Panel B) along with the 95% confidence interval in parentheses. The table reports a medium-term exposure effect defined as exposure to pollution over the semester with 6 months lag. Covariates are at individual, household and neighbourhood levels listed in Table 1. * p < 0.05, ** p < 0.01, *** p <0.001.*

Supplementary Table 19. Hazard ratios (95% CI) for the association between medium-term exposure to $PM_{2.5}$ and NO_2 and Parkinson's disease, overall sample, 18 months lag exposures, by age

	Age in 2011 < 50 years		Age in 2011 >= 50 years	
	Model 1	Model 2	Model 1	Model 2
Panel A ($PM_{2.5}$, μg/m^3)				
Linear model	1.11***	1.05*	1.00	0.97
	(1.07 -1.16)	(1.00 -1.10)	(0.96-1.03)	(0.93-1.00)
Quartiles model				
1st (reference)				
2nd	1.18	1.16	0.94	0.93
	(0.98-1.89)	(0.91-1.48)	(0.81-1.09)	(0.82-1.09)
3rd	1.36*	1.31*	0.94	0.92
	(1.07-1.73)	(1.03-1.67)	(0.81-1.09)	(0.79-1.07)
4th	1.54***	1.27	0.96	0.88
	(1.21-1.97)	(1.01-1.67)	(0.82-1.12)	(0.74-1.04)
Observations	1409732	1409732	1271678	1271678
Covariates	NO	YES	NO	YES
Panel B (NO_2, μg/m^3)				
Linear model	1.02***	1.01	0.99	0.99*
	(1.01-1.04)	(0.99-1.02)	(0.99-1.00)	(0.98-1.00)
Quartiles model				
1st (reference)				
2nd	1.06	1.06	1.01	1.01
	(0.82-1.24)	(0.81-1.24)	(0.89-1.14)	(0.89-1.15)
3rd	1.31**	1.24*	0.92	0.90
	(1.08-1.60)	(1.00-1.54)	(0.81-1.04)	(0.78-1.02)
4th	1.41***	1.20	0.95	0.86*
	(1.15-1.72)	(0.94-1.51)	(0.83-1.07)	(0.75-1.00)
Observations	1409732	1409732	1271678	1271678
Covariates	NO	YES	NO	YES

*Notes. Each model is a Cox Proportional Hazard (CPH) model with standard error clustered by SOA. Sample splits by age below and above 50, and each cell presents the estimated hazard ratio for a 1 μg/m3 increase in PM2.5 (Panel A) and NO2 (Panel B) along with the 95% confidence interval in parentheses. The table reports a medium-term exposure effect defined as exposure to pollution over the semester with 18 months lag. Covariates are at individual, household and neighbourhood levels listed in Table 1. * p < 0.05, ** p < 0.01, *** p <0.001.*

Supplementary Table 20. Hazard ratios (95% CI) for the association between 2-year moving average exposure to $PM_{2.5}$ and NO_2 and Parkinson's Disease onset, one year lag, by age

	Age in 2011 < 50 years		Age in 2011 >= 50 years	
	Model 1	Model 2	Model 1	Model 2
Panel A ($PM_{2.5}$, µg/m³)				
Linear model	1.12***	1.06*	0.99	0.96*
	(1.07 -1.17)	(1.01 -1.11)	(0.95-1.02)	(0.92-0.99)
Quartiles model				
1st (reference)				
2nd	0.96	0.99	0.94	0.97
	(0.77-1.19)	(0.78-1.24)	(0.82-1.09)	(0.84-1.12)
3rd	1.14	1.13	0.97	0.96
	(0.92-1.41)	(0.90-1.42)	(0.85-1.12)	(0.83-1.12)
4th	1.49***	1.28*	0.93	0.86*
	(1.22-1.82)	(1.02-1.61)	(0.81-1.07)	(0.74-0.99)
Observations	1386259	1386259	1253363	1253363
Covariates	NO	YES	NO	YES
Panel B (NO_2, µg/m³)				
Linear model	1.03***	1.00	0.99	0.99*
	(1.01-1.04)	(0.99-1.02)	(0.99-1.00)	(0.97-1.00)
Quartiles model				
1st (reference)				
2nd	0.98	0.98	1.01	1.02
	(0.79-1.22)	(0.78-1.22)	(0.82-1.06)	(0.89-1.16)
3rd	1.18	1.12	0.91	0.90
	(0.95-1.45)	(0.89-1. 04)	(0.78-1.01)	(0.78-1.02)
4th	1.45***	1.24	0.95	0.88
	(1.19-1.18)	(0.99-1.56)	(0.84-1.09)	(0.76-1.01)
Observations	1386247	1386247	1253361	1253361
Controls	NO	YES	NO	YES

Notes. Each model is a Cox Proportional Hazard (CPH) model with standard error clustered by SOA. Sample splits by age below and above 50, and each cell presents the estimated hazard ratio for a 1 µg/m3 increase in PM2.5 (Panel A) and NO2 (Panel B) along with the 95% confidence interval in parentheses. The table reports 2 years moving average exposure effect with one year lag. Covariates are at individual, household and neighbourhood levels listed in Table 1.
** p < 0.05, ** p < 0.01, *** p <0.001.*

Supplementary Table 21. Hazard ratios (95% CI) for the association between medium-term exposure to $PM_{2.5}$ and NO_2 and Parkinson's disease, overall sample, with one year lag, extended at-risk period, by age

	Age in 2011 < 50 years		Age in 2011 >= 50 years	
	Model 1	Model 2	Model 1	Model 2
Panel A ($PM_{2.5}$, µg/m³)				
Linear model	1.12***	1.05*	1.00	0.97
	(1.07 -1.16)	(1.00 -1.10)	(0.97-1.03)	(0.94-1.01)
Quartiles model				
1st (reference)				
2nd	1.31*	1.24*	0.97	0.96
	(1.06-1.60)	(1.01-1.53)	(0.86-1.10)	(0.85-1.09)
3rd	1.28*	1.19	0.94	0.91
	(1.03-1.60)	(0.95-1.49)	(0.82-1.07)	(0.79-1.04)
4th	1.60***	1.27*	1.01	0.92
	(1.29-1.99)	(1.00-1.60)	(0.88-1.17)	(0.79-1.07)
Observations	1693748	1693748	1541026	1541026
Covariates	NO	YES	NO	YES
Panel B (NO_2, µg/m³)				
Linear model	1.02***	1.00	0.99	0.99*
	(1.01-1.04)	(0.99-1.02)	(0.99-1.01)	(0.98-1.00)
Quartiles model				
1st (reference)				
2nd	1.05	1.04	0.99	0.98
	(0.86-1.27)	(0.86-1.27)	(0.88-1.11)	(0.87-1.10)
3rd	1.28*	1.20	0.92	0.88*
	(1.06-1.54)	(0.98-1.47)	(0.81-1.03)	(0.78-1.00)
4th	1.39***	1.16	0.98	0.86*
	(1.14-1.68)	(0.93-1.44)	(0.85-1.11)	(0.76-0.98)
Observations	1693748	1693748	1541026	1541026
Covariates	NO	YES	NO	YES

*Notes. Each model is a Cox Proportional Hazard (CPH) model with standard error clustered by SOA. Sample splits by age below and above 50, and each cell presents the estimated hazard ratio for a 1 µg/m3 increase in PM2.5 (Panel A) and NO2 (Panel B) along with the 95% confidence interval in parentheses. The table reports a medium-term exposure effect defined as exposure to pollution over the semester with one year lag. Extended at-risk sample refers to extending at risk period to 2010 S2. Covariates are at individual, household and neighbourhood levels listed in Table 1. * p < 0.05, ** p < 0.01, *** p <0.001.*

***Supplementary* Table 22. Hazard ratios (95% CI) for the association between medium-term exposure to PM2.5 and NO2 and Parkinson's Disease onset, one year lag, by age with alternative definition of PD**

	Age in 2011 < 50 years		Age in 2011 >= 50 years	
	Model 1	Model 2	Model 1	Model 2
Panel A ($PM_{2.5}$, μg/m³)				
Linear model	1.13***	1.06	0.99	0.96
	(1.06 -1.20)	(0.99 -1.14)	(0.95-1.03)	(0.92-1.00)
Quartiles model				
1st (reference)				
2nd	0.98	0.95	1.04	1.04
	(0.71-1.34)	(0.69-1.31)	(0.87-1.25)	(0.86-1.25)
3rd	1.21	1.15	0.97	0.94
	(0.88-1.65)	(0.83-1.60)	(0.80-1.17)	(0.78-1.15)
4th	1.52**	1.23	1.03	0.93
	(1.11-2.09)	(0.87-1.75)	(0.85-1.26)	(0.76-1.14)
Observations	1415122	1415122	1277060	1277060
Covariates	NO	YES	NO	YES
Panel B (NO_2, μg/m³)				
Linear model	1.03**	1.01	1.00	0.99
	(1.01-1.05)	(0.99-1.03)	(0.98-1.00)	(0.97-1.00)
Quartiles model				
1st (reference)				
2nd	0.79	0.79	0.91	0.91
	(0.58-1.08)	(0.57-1.10)	(0.77-1.08)	(0.77-1.08)
3rd	1.26	1.22	0.94	0.91
	(0.96-1.66)	(0.89-1.66)	(0.79-1.10)	(0.76-1.08)
4th	1.45**	1.25	0.93	0.83
	(1.10-1.92)	(0.90-1.75)	(0.79-1.10)	(0.70-1.00)
Observations	1415122	1415122	1277060	1277060
Covariates	NO	YES	NO	YES

*Notes. Each model is a Cox Proportional Hazard (CPH) model with standard error clustered by SOA. Each cell presents the estimated hazard ratio for a 1 μg/m3 increase in PM2.5 (Panel A) and NO2 (Panel B) along with the 95% confidence interval in parentheses. Parkinson disease defines as subjects receiving at least two prescriptions in two consecutive semesters. The table reports a medium-term exposure effect defined as exposure to pollution over the semester with one year lag. Covariates are at individual, household and neighbourhood levels listed in Table 1. * p < 0.05, ** p < 0.01, *** p <0.001.*

Supplementary Table 23. BNF codes and names of drugs used as PD indicators

Parkinsonism (BNF Chapter 4, Section 9)		
Park_4_9	=1 if prescribed any item whose BNF code begins with the following 4 characters: 0409	4.9 includes three sub-sections:<br>1) Dopaminergic (dopamine) drugs used to treat Parkinsonism;<br>2) Antimuscarinic Drugs Used In Parkinsonism;<br>3) Essential tremor, Chorea, Tics & Related disorders.
